# Re-Irradiation for Recurrent Head and Neck Cancer: Freedom from Cancer Recurrence Rate

**DOI:** 10.3390/jcm12082979

**Published:** 2023-04-19

**Authors:** Issa Mohamad, Taher Abu Hejleh, Sania Abdelqader, Lina Wahbeh, Ayat Taqash, Abdelatif Almousa, Ebrahim Mayta, Akram Al-Ibraheem, Fawzi Abuhijla, Ramiz Abu-Hijlih, Tariq Hussein, Wisam Al-Gargaz, Hamza Ghatasheh, Ali Hosni

**Affiliations:** 1 Department of Radiation Oncology, King Hussein Cancer Center, Amman 11942, Jordan; sa.14752@khcc.jo (S.A.); lw.15102@khcc.jo (L.W.); aalmousa@khcc.jo (A.A.); fhijle@khcc.jo (F.A.);; 2 Department of Medical Oncology, King Hussein Cancer Center, Amman 11942, Jordan; ta.11703@khcc.jo; 3 Department of Biostatistics, King Hussein Cancer Center, Amman 11942, Jordan; ataqash@khcc.jo; 4 Department of Surgical Oncology, King Hussein Cancer Center, Amman 11942, Jordan; emayta@khcc.jo (E.M.);; 5 Department of Nuclear Medicine, King Hussein Cancer Center, Amman 11942, Jordan; aibraheem@khcc.jo; 6 Radiation Medicine Program, Princess Margaret Cancer Centre, University Health Network, University of Toronto, Toronto, ON M5G 2M9, Canada

**Keywords:** re-irradiation, head and neck, cancer, outcomes

## Abstract

Salvage re-irradiation (rRT) for patients with locoregionally recurrent head and neck cancer (rHNC) remains challenging. A retrospective analysis was performed on 49 patients who received rRT between 2011 and 2018. The co-primary endpoint of the study was 2-year freedom from cancer recurrence rate (FCRR) and overall survival (OS), and secondary endpoints were 2-year disease-free survival (DFS), local failure (LF), regional failure (RF), distant metastases (DM), and RTOG grade 3 ≥ late toxicities. Adjuvant and definitive rRT were delivered to 22 and 27 patients, respectively. A total of 91% of patients were managed with conventional re-RT and 71% of patients received concurrent chemotherapy. The median follow-up after rRT was 30 months. The 2-year FCRR, OS, DFS, LF, RF, and DM were 64%, 51%, 28%, 32%, 9%, and 39% respectively. MVA showed that poor performance status (PS: 1–2 vs. 0) and age > 52 years were predictive of worse OS. In comparison, poor PS (1–2 vs. 0) and total dose of rRT < 60 Gy were predictive of worse DFS. Late RTOG toxicity of grade 3 ≥ was reported in nine (18.3%) patients. FCRR at 2 years after salvage rRT for rHNC was higher than other traditional endpoints and could be an important endpoint to be included in future rRT studies. rRT for rHNC at our cohort was relatively successful, with a manageable level of late severe toxicity. Replacing this approach in other developing countries is a viable option.

## 1. Introduction

Approximately 17–30% of patients with head and neck cancer (HNC) would have locoregional recurrence (LRR) following curative-intent treatment [1]. Palliative chemotherapy with or without targeted therapy or immunotherapy resulted in non-curable short-term responses with relatively short survival [2,3,4,5]. Whenever feasible (based on available resources, expertise, patient, and tumor characteristics at time of LRR), the use of potentially curative locoregional treatment such as salvage surgery and adequate dose re-irradiation (rRT)+/− concurrent chemotherapy is encouraged [6,7,8,9,10].

Management of LRR arising in a previously irradiated volume has always been a complex clinical and dosimetric situation in the context of old radiation techniques. The Radiation Therapy Oncology Group (RTOG) 9610 and 9911 had reported a higher rate of severe rRT-related morbidity, with approximately 8% of patients experiencing treatment-related deaths (for different reasons, e.g., fatal hemorrhage, febrile neutropenia, dehydration, shock, and pneumonitis). The use of historical rRT techniques for recurrent head and neck cancer (rHNC) was associated with low survival rates, with a 2-year overall survival rate (OS) of 15–26% [11,12]. However, a large multi-institutional cohort study using newer rRT such as intensity-modulated radiation therapy (IMRT) showed a relatively low rate of grade 4 acute toxicity (5.1%) and a higher 2-year OS (35.4%) compared to previously reported historical data [9,13].

Morbidity in rHNC is primarily caused by cancer recurrence and/or treatment-related toxicity. The advantages of rRT should outweigh the associated risks of such treatment. Patients require a multidisciplinary evaluation to determine the best management strategy [13,14]. Salvage surgery is the preferred choice for resectable cases, either with or without adjuvant rRT (+/− concurrent chemotherapy) as indicated by postoperative histopathological findings. Unfortunately, many recurrences present in the advanced T- and N-categories and are not surgically resectable. In unresectable or medically inoperable cases, definitive rRT with or without concurrent chemotherapy is the appropriate treatment option in carefully selected patients [9,11,13]. rRT can be administered via IMRT, stereotactic body radiation therapy (SBRT) whenever possible, or proton therapy whenever available [9,15,16].

The previous studies mainly focused on traditional outcomes such as OS, DFS, LF, RF, and DM. However, these endpoints do not adequately reflect the success of salvage rRT. Hence, in this study, we assessed the freedom from cancer recurrence rate (FCRR), which evaluates the success of salvage rRT. Additionally, we present the traditional oncologic outcomes of rRT for patients with rHNC at our institution.

## 2. Materials and Methods

### 2.1. Study Population

After institutional research board (IRB) approval (IRB No. 21 KHCC 189), we identified all patients with rHNC who had pathologically confirmed non-metastatic recurrent squamous cell carcinoma (SCC) of the head and neck (H&N) and who had previously received RT to the H&N region at a total dose of ≥44 Gy. These patients were staged according to the seventh edition of the TNM staging system jointly used by the American Join Committee on Cancer (AJCC) and Union of the International Cancer Control (UICC). Patients salvaged with curative-intent rRT at our institution between 2011 and 2018 were included in this retrospective analysis. Patients younger than 18 years, and those with histopathology other than SCC were excluded from this analysis. The patients’ demographics and clinical information including outcomes were retrospectively collected from the patients’ medical records.

### 2.2. Diagnostic Approach

Re-staging work up and pre-salvage treatment evaluation consisted of a comprehensive physical examination including assessment with fiberoptic endoscopy. Imaging evaluation included H&N MRI and PET/CT scans. After completion of re-staging work up, all patients were discussed and managed by a multidisciplinary H&N team, with evaluation by dedicated teams of dental oncologists, nutritionists, and speech/language pathologists prior to the initiation of rRT.

### 2.3. Treatment Approach

All patients who developed local and/or regional recurrence were evaluated by a HN surgeon for possible salvage surgery. Adjuvant rRT was considered for pT3/4, close resection margin(s), and/or multiple involved lymph nodes, while adjuvant concurrent chemotherapy (with rRT) was considered for involved resection margin(s) and/or pathologic extranodal extension (pENE). Patients with unresectable tumors were managed with definitive rRT with or without concurrent chemotherapy.

The dose and fractionation schedules of the rRT regimens were selected based on institutional guidelines. In general, conventional fractionation (1.8–2 Gy/fraction) rRT was used with a minimum total prescribed dose of 44 Gy and a maximum total prescribed dose of 70 Gy (whenever possible) according to: (1) intent of rRT (adjuvant vs. definitive), (2) the degree of overlap with previous radiation, (3) proximity to critical organs-at-risk (i.e., neuro-ocular structures), and (4) time interval between the two courses of radiation. At the discretion of the treating radiation oncologist (after a comprehensive review of the old radiation plan), a hyperfractionation rRT schedule (for more complex cases) or a hypofractionation rRT regimen (for less complex cases with small volume tumors far away from critical structures) could be used. rRT was delivered using IMRT. Concurrent chemotherapy (in the definitive or adjuvant setting) consisted of weekly cisplatin (40 mg/m^2^ weekly) or carboplatin (area under the curve [AUC] of 1.5 weekly) during rRT.

### 2.4. Post-Treatment Evaluation and Follow-Up

In general, patients were reviewed in the radiation oncology clinic 2 weeks after the end of rRT, then every 3 months for the first 2 years, every 4 months in the third year, every 6 months in the fourth and fifth year, and annually thereafter until death. Post-treatment imaging to evaluate the response to therapy included H&N MRI and PET/CT scans, which were performed 10–12 weeks after the end of rRT, then as clinically indicated. Severe late rRT-related side effects were defined as late RTOG grade ≥ 3 toxicity starting > 6 months after the end of rRT.

### 2.5. Statistical Methods

The co-primary endpoint of the study was 2-year FCRR and OS, and secondary endpoints were 2-year local failure (LF), regional failure (RF), distant metastases (DM), disease-free survival (DFS), and late toxicities. OS and DFS were analyzed using the Kaplan–Meier method and compared using the log-rank test. LF, RF, and DM rates were estimated using the cumulative incidence method using Fine Gray’s test, with death (without disease recurrence) as a competing risk. FCRR was estimated by the competing risk method (LF, RF, and DM are events, while death without LF, RF, and DM was considered a competing factor). Late toxicity rates were estimated by the cumulative incidence function. Multivariable analysis (MVA) using Cox proportional hazards regression was used to identify predictors of OS and DFS. All reported *p*-values were two-sided, with a statistical significance level of *p* ≤ 0.05. All analyses were performed using SAS version 9.4 (SAS Institute Inc., Cary, NC, USA), and the figures were created using GraphPad PRISM 7.

## 3. Results

### 3.1. Patient, Tumor, and Treatment Characteristics

Patients, tumor, and treatment characteristics for the whole cohort, definitive rRT, and rRT adjuvant groups are summarized in Table 1.

A total of 49 rHNC patients [nasopharyngeal (*n* = 12); laryngeal (*n* = 12); oral cavity (*n* = 11); salivary gland (*n* = 9); and skin (*n* = 5)] were identified, of whom 22 patients (45%) were treated with adjuvant rRT with (*n* = 14) or without (*n* = 8) concurrent chemotherapy, while 27 patients (55%) were managed with definitive rRT with (*n* = 20) or without (*n* = 7) concurrent chemotherapy. The median (range) age at the time of recurrence for the whole cohort was 53 (21–80) years. Approximately half of the patients (51%) had performance status (PS) 0, while the remaining had PS 1–2.

Conventional fractionation rRT (44–70 Gy with 1.8–2 Gy/fraction) was used in forty-five patients [nasopharyngeal (*n* = 12); laryngeal (*n* = 10); oral cavity (*n* = 10); salivary gland (*n* = 9); and skin (*n* = 4)], hyperfractionation with 1.1 Gy/fraction twice daily to 44–63.8 Gy was used in three patients [laryngeal (*n* = 2) and oral cavity (*n* = 1)], and hypofractionation with 2.5 Gy/fraction to 50 Gy was used in one patient with recurrent skin cancer. There were no statistically significant differences between the treatment groups (adjuvant vs. definitive rRT) except for the recurrent tumor location (*p* ≤ 0.01) and rRT fractionation schedule (*p* < 0.04) as shown in Table 1.

### 3.2. Survival Outcomes

**Figure 1:**
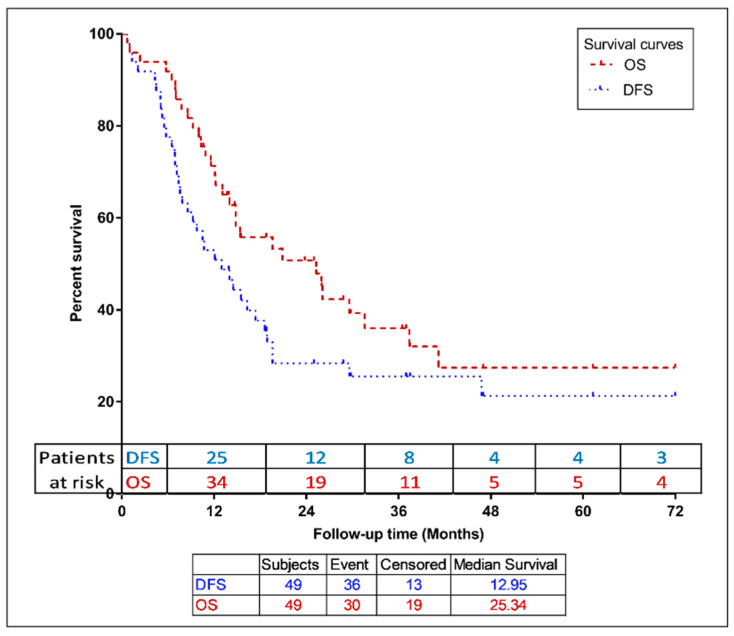
Kaplan–Meier curve for overall and disease-free survival in the entire study population. OS, overall survival; DFS, disease free survival.

For the entire cohort, the 2-year OS and DFS were 51% (95% CI, 36–65%) and 28% (95% CI, 16–42%) and the 5-year OS and DFS were 27% (95% CI, 14–44%) and 21% (95% CI, 10–36%), respectively (Figure 1).

There was no statistically significant difference in the 2- and 5-year OS rates between the adjuvant rRT vs. definitive rRT groups; (2-year OS: 53% [95% CI, 32–74%] vs. 49% [95% CI, 30–68%, *p* = 0.55] and 5-year OS: 20% [95% CI, 3.5–46%] vs. 33% [95% CI, 15–54%, *p* = 0.55]), respectively. There was no statistically significant difference in the 2- and 5-year DFS rates between the adjuvant rRT vs. definitive rRT groups; (2-year DFS: 24% [95% CI, 8–45%] vs. 32% [95% CI, 16–51%, *p* = 0.8] and 5-year DFS: 16 [95% CI, 3–37%] vs. 25% [95% CI, 10–45%, *p* = 0.8]), respectively.

### 3.3. Tumor Control Outcomes

**Figure 2:**
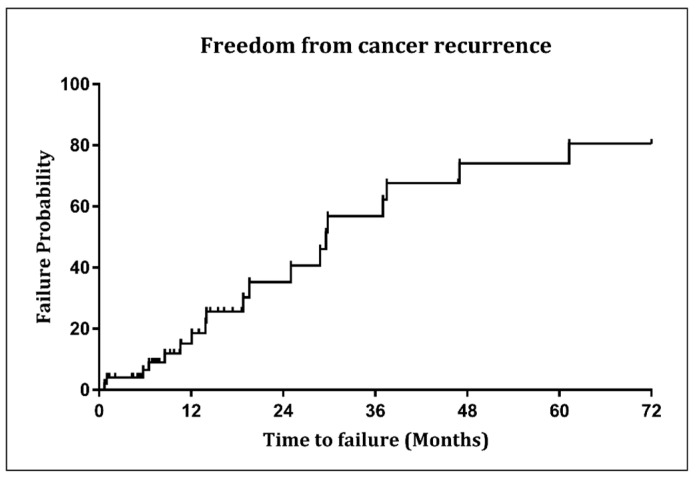
Cumulative incidence method for freedom from cancer recurrence rate in the entire study population.

The 2- and 5-year FCRR values for the entire cohort were 64% (95% CI 45–81) and 26% (9–47), respectively, for the definitive RT group were 71% (95% CI 46–91) and 33% (95% CI 10–63), respectively, and for the adjuvant rRT group were 59% (95% CI 32–84) and 15% (95% CI 8–48), respectively (Figure 2).

**Figure 3:**
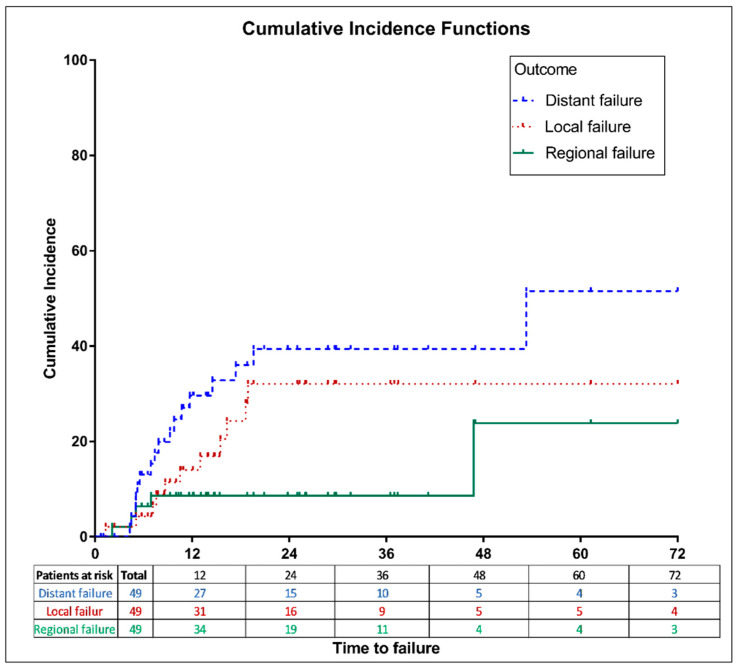
Cumulative incidence method for local, regional, and distant failures in the entire study population.

For the entire cohort, the 2-year LF, RF, and DM cumulative incidence rates were 32% (95% CI, 16–48), 9% (95% CI, 3–19), and 39% (95% CI, 24–55), respectively, and the 5-year LF, RF, and DM cumulative incidence rates were 32% (95% CI, 16–48), 24% (95% CI, 3–56), and 52% (95% CI, 23–74), respectively, as shown in Figure 3.

For patients who developed LF (*n* = 11) and RF (*n* = 5), the delivered BED_10_ at the time of rRT ranged between 34–80 Gy_10_ as shown in Table 2.

Eleven patients developed LF (adjuvant rRT: *n* = 6 vs. definitive rRT: *n* = 5) at the median time of 13.7 (range: 7–41) months after rRT. Of whom, one patient had synchronous RF and three patients had DM at the time of LF, and were subsequently treated with palliative chemotherapy.

Five patients developed RF (adjuvant rRT: *n* = 1 vs. definitive rRT: *n* = 4) at the median time of 7 (range: 2–72) months post-rRT. Of whom, one patient had LF and three patients had DM at the time of RF, and were treated with palliative chemotherapy.

Seventeen patients developed DM (adjuvant rRT: *n* = 7 vs. definitive rRT: *n* = 10) at the median time of 14.8 (range: 7–72) months post-rRT. Of whom, three patients had DM with LF and three patients had DM with RF, while eleven patients developed distant-only failure. The most common sites of DM were lung (*n* = 5, 29%) and bone (*n* = 5, 29%). Other sites of DM were brain (*n* = 3, 18%), liver (*n* = 3, 18%), and skin (*n* = 1, 6%). All metastatic patients were treated with palliative chemotherapy. The median time from DM to death was 2.5 months (range, 0.3–18.3).

### 3.4. Toxicity Outcomes

The grade 3 ≥ late RTOG toxicity was reported in nine (18.3%) patients. This included grade 3 dysphagia (*n* = 5), which necessitated the placement of a feeding tube 6 months after the end of rRT, grade 3 osteoradionecrosis (*n* = 3), grade 3 brain necrosis (*n* = 2), grade 3 neck fibrosis (*n* = 2), and grade 5 carotid blowout (*n* = 1). Out of 49 patients studied, 10 (20.4%) patients experienced grade 2 RTOG toxicity related to late dysphagia, while 20 (40.8%) patients experienced grade 2 RTOG toxicity related to late xerostomia. The cumulative prescribed BED_10_ for patients who developed grade 3 ≥ late RTOG toxicity ranged between 91 Gy_10_ and 121 Gy_10_ as shown in Table 3.

The patient, who developed carotid blowout, was a 57-year-old at the time of diagnosis of a recurrent unresectable regional recurrence on the background of previously irradiated T1N0M0 glottic cancer (originally received 63 Gy in 28 fractions using conventional technique with two opposing lateral fields). The unresectable neck recurrence involved the right neck nodal levels II-IV, the cumulative BED 10 was 98 Gy_10_, and the interval between original RT and rRT was 18 months. 

### 3.5. Outcome Predictors

On MVA, PS (1–2 vs. 0) (HR, 3.53; 95% CI, 1.52–8.19, *p* = 0.01) and age > 52 years at time of recurrence (HR, 5.122; 95% CI, 1.901–13.804, *p* = 0.01) years predicted worse OS. However, PS (1–2 vs. 0; HR, 2.800; 95% CI, 1.402–5.590, *p* = 0.01) and rRT dose (<60 Gy vs. ≥60 Gy; HR, 0.341; 95% CI, 0.126–0.919, *p* = 0.033) predicted worse DFS as seen in Table 4.

## 4. Discussion

This paper introduces FCRR as an endpoint for rRT in rHNC. To our knowledge, this endpoint was not used previously in clinical trials. The significance of FCRR lies in the fact that part of the primary cause of morbidity in rHNC is cancer recurrence and treatment-related toxicity. Previously, death was used as an event in calculating DFS, potentially minimizing the impact of cancer recurrence on morbidity and quality of life (QoL). FCRR, on the other hand, focuses solely on the cancer recurrence rate. Our study found that 64% of patients had a FCRR at 2 years after rRT, which was higher than other outcomes such as OS, DFS, LF, RF, and DM. The relationship between FCRR and QoL in rHNC patients requires further exploration. This study indicates a substantial proportion of non-metastatic rHNC patients can achieve disease control and survival after rRT using the IMRT technique. These findings align with the results of previous studies [9,10,17,18,19]. In a study by the University of Texas MD Anderson Cancer Center, they reported that after 2 years of receiving rRT with IMRT, 58% and 64% of 78 patients achieved OS and locoregional control, respectively [19]. Lee et al. reported 2-year PFS and OS rates of 30.9% and 54.6%, respectively, in 42 HNC patients who underwent salvage rRT for non-metastatic locoregional recurrence [18].

This study found that there was no significant difference in OS between patients who received adjuvant or definitive rRT for rHNC. This suggests that salvage surgery did not provide a survival benefit for this patient population. The study’s outcome contradicts earlier research by the Multi-institution Radiotherapy Collaborative Group (MIRI), which showed a 2-year OS rate of 45% and 36% for postoperative and definitive rRT, respectively [13]. The Korean Radiation Oncology Group, in the study (KROG 1707), also reported that salvage surgery for rHNC was associated with superior OS (*p* = 0.002) [17]. The difference in results may be due to several factors including: optimal patient selection at a specialized cancer center, a larger number of patients in our study with NPC (known to be highly responsive to radiotherapy and chemotherapy, and high salvage success rates with rRT) [20,21], and more advanced T4-category among patients who underwent surgery compared to those who received definitive rRT (55% *n* = 12 vs. 33% *n* = 9, respectively). Furthermore, too many treatment regimens were used in our study that weaken the generalizability of our rRT endpoints. However, the data regarding rRT is sparse in the literature. Several studies show institutional experience of relatively very small numbers of patients [18,19]. There is no consensus recommendation regarding the actual regimen that can be used16. In addition, every case represents a challenging situation when constraints for critical organs at risk can be met. All these reasons make it different to generalize the conclusion for retrospective studies of rRT (including our study); however, it provides some practical guidance for managing rHNC cases.

In this study, the factors that were found to be associated with better OS included good PS (ECOG 0) and young age (below 52 years). Furthermore, the study results showed that improved DFS was observed in patients who had a PS of ECOG 0 and received a rRT dose > 60 Gys [14]. A higher radiotherapy dose was typically associated with improved local control of rHNC. Our results were similar to Roesch et al. who conducted a large multicenter analysis of dose-escalated rRT for rHNC in Germany. His study included 253 patients treated at 16 university hospitals. The results showed that patients with good ECOG PS and rRT doses above 50 Gy (EQD2) had median longer OS (17.8 months vs. 11.7 months, *p* < 0.01) and PFS (9.6 months vs. 6.8 months, *p* < 0.01) compared to those with poor ECOG PS and rRT below 50 Gy (EQD2) (*p* < 0.01) [22]. These findings provide important information for clinicians who are considering rRT treatment options for patients with rHNC [9,13,14].

Although severe late toxicity from salvage treatment of rHNC is a major concern, not providing treatment can also have severe consequences, as rHNC can cause significant morbidity and mortality [3]. In our study population, severe grade 3 ≥ late toxicity was seen in 18.3% of the cases, which is comparable to the 16.3% rate reported by the MIRI collaborative group [23]. These findings emphasize the importance of carefully weighing the potential benefits and risks of salvage rRT for rHNC.

The limitations of this paper include its retrospective design, limited sample size, and the diverse primary site locations of the rHNC tumors. Despite our findings, it is advisable to exercise caution when promoting the use of salvage rRT for rHNC patients. A multidisciplinary approach to managing rHNC remains the recommended course of action in these complex cases.

## 5. Conclusions

The 2-year FCRR after salvage rRT for rHNC was superior to other traditional endpoints. Given that the morbidity of rHNC is largely linked to locoregional recurrence regardless of survival, FCRR may serve as a new endpoint to consider in rRT studies for rHNC. For studies with a small sample size, FCRR may serve as a more suitable endpoint. The rate of late severe toxicity was acceptable.

## Figures and Tables

**Table 1:** Patients, tumor, and treatment characteristics.

Variable		Whole Cohort N = 49 (100%)	Sub-Groups		*p* Value
			Adjuvant rRT N = 22 (45%)	Definitive rRT N = 27 (55%)	
Follow up, median (range), months, all patients		29.8 (10.6–72)	24.4 (13.9–138)	37.5 (10.6–109)	0.32
Age, median (range), years		53 (21–80)	53 (27–68)	54 (21–80)	0.93
Gender	Female	13 (27%)	6 (27%)	7 (26%)	0.91
	Male	36 (73%)	16 (73%)	20 (74%)	
PS	0	25 (51%)	14 (64%)	11 (41%)	0.11
	1–2	24 (49%)	8 (36%)	16 (59%)	
rT-category	rT0–2	24 (49%)	9 (41%)	15 (56%)	0.308
	rT3–4	25 (51%)	13 (59%)	12 (44%)	
rN-category	rN0	31 (63%)	14 (64%)	17 (63%)	0.96
	rN1–3	18 (37%)	8 (36%)	10 (37%)	
Recurrent tumor site	Larynx	12 (25%)	6 (27.3%)	6 (22.2%)	**0.01**
	NPC	12 (25%)	-	12 (44.5%)	
	Oral cavity	11 (22%)	8 (36.3%)	3 (11.1%)	
	Salivary gland	9 (18%)	4 (18.2%)	5 (18.5%)	
	Skin	5 (10%)	4 (18.2%)	1 (3.7%)	
Time since prior RT	≤2 years >2 years	11 (22%) 38 (78%)	5 (22.7%) 17 (77.3%)	6 (22.2%) 21 (77.8%)	0.96
Time since prior RT, median (range), months		39.5 (11–238)	42 (11–238)	35 (16- 234)	0.55
rRT BED_10_, median (range), Gy_10_		56.5 (34–80)	55 (34–78)	57 (34–80)	0.37
Cumulative-BED_10_, median (range), Gy_10_		84 (86–149)	112 (94–149)	115 (86–136)	0.37
rRT fractionation schedule	Conventional Others	45 4	18 (82%) 4 (18%)	27 (100%) -	**0.04**
rRT total dose (Gy)	≤60 >60	38 11	18 (82%) 4 (18%)	20 (74%) 7 (26%)	0.73
Concurrent chemotherapy	No Yes	14 35	8 (36%) 14 (64%)	6 (22%) 21 (78%)	0.28
Type of weekly concurrent chemotherapy	Cisplatin Carboplatin	13 22	6 (43%) 8 (57%)	7 (33%) 14 (67%)	0.57

Significant *p*-values in bold. PS, performance status; rRT, re-irradiation; BED, biological equivalent dose; NPC, nasopharyngeal carcinoma.

**Table 2:** The recurrent tumor site and stage, treatment delivered at the time of recurrence, rRT dose fractionation schedule used at the time of rRT, cumulative BED_10_, and patterns of recurrence after rRT.

Patient	Site of Locoregional Recurrence at the Time of rRT	rTNM	Treatment Delivered	Planned rRT Dose/Fractionation (Delivered Dose)	Cumulative BED_10_	Local Recurrence after rRT	Regional Recurrence after rRT
1	Skin/nasal vestibule (local)	rpT2N0M0	Adjuvant rRT	60 Gy/30 frs	110 Gy	Yes	No
2	Skin/nasal vestibule (local)	rpT2N0M0	Adjuvant rRT	50 Gy/20 frs	102 Gy	Yes	No
3	Larynx (local)	rpT2N0M0	Adjuvant rRT	59.4 Gy/54 frs (BID)	110 Gy	Yes	No
4	Oral cavity/buccal (local)	rpT4aN0M0	Adjuvant CCRT	66 Gy/33 frs	111 Gy	Yes	No
5	Oral cavity/tongue (local)	rpT4aN0M0	Adjuvant CCRT	60 Gy/30 frs (delivered 40 Gy in 20 frs)	92 Gy	Yes	No
6	Oral cavity (tongue) (local)	rpT4aNM0	Adjuvant CCRT	60 Gy/30 frs (delivered 28 Gy in 14 frs)	74 Gy	Yes	No
7	Oral cavity (tongue)/neck (bilateral levels II–III) (regional)	rT0N3M0	Adjuvant rRT	60 Gy/30 frs	117 Gy	No	Yes
1	Oral cavity (tongue)/neck left level II (locoregional)	rT3N1M0	Definitive CCRT	66 Gy/33 frs	136 Gy	Yes	Yes
2	Nasopharynx (local)	rT3N0M0	Definitive CCRT	44 Gy/22 frs	108 Gy	Yes	No
3	Nasopharynx (local)	rT3N0M0	Definitive CCRT	50 Gy/25 frs	112 Gy	Yes	No
4	Nasopharynx (local)	rT1N0M0	Definitive CCRT	60 Gy/30 frs	118 Gy	Yes	No
5	Nasopharynx (local)	rT1N0M0	Definitive RT	54 Gy/27 frs	109 Gy	Yes	No
6	Neck (left level III) (regional)	rT0N3M0	Definitive CCRT	66 Gy/33 frs	121 Gy	No	Yes
7	(Right level III) (regional)	rT0N2aM0	Definitive CCRT	70 Gy/35 frs	112 Gy	No	Yes
8	Left parotid SCC (regional)	rT0N2aM0	Definitive CCRT	66 Gy/33 frs	117 Gy	No	Yes

rRT; re-irradiation; BED_10_; biological equivalent dose; CCRT, concurrent chemoradiation; SCC, squamous cell carcinoma; rTNM, Recurrent tumor, nodal and metastasis; BID, twice a day.

**Table 3:** The primary tumor site, recurrent tumor site, recurrent rTN-category, time interval since prior RT treatment, grade 3 ≥ late RTOG toxicity, time interval to development of grade 3 ≥ toxicity, rRT BED_10_, dose fractionation schedule used at the time of rRT, rRT BED_10_, and cumulative BED_10_.

Patient	Primary Tumor Site	Recurrent Tumor Site	Recurrent TN-Category	Time Interval since Prior RT	Grade 3 ≥ Late RTOG	Time Interval to Development of Grade 3 ≥ Toxicity after rRT	rRT BED_10_	Cumulative BED_10_
Adjuvant rRT/CCRT								
1	Larynx	Larynx	rpT2N0	45 months	Dysphagia and neck fibrosis grade 3	24 months	62 Gy	121 Gy
2	Left submandibular gland	Left neck level II	rpT0N3	36 months	Dysphagia grade 3	24 months	62 Gy	121 Gy
3	Oral cavity	Tongue/right level II	rpT4aN1	39 months	Dysphagia grade 3 and osteoradionecrosis	21 months	60 Gy	111 Gy
4	Oral cavity	Tongue	rpT4aN0	22 months	Osteoradionecrosis	9 months	61 Gy	121 Gy
5	Oral cavity	Tongue (perinural recurrence-V3)	rpT4aN0	33 months	Brain necrosis	7 months	44 Gy	100 Gy
Definitive rRT/CCRT								
6	Larynx	Larynx and left level III	rT4aN1	19 months	Dysphagia and neck fibrosis grade 3	27 months	55 Gy	116 Gy
7	Oral cavity	Left buccal	rT4aN0	26 months	Osteoradionecrosis and dysphagia grade 3	4 months	57 Gy	115 Gy
8	Larynx	Larynx and bilateral levels II-IV	rT3N3	40 months	Carotid blowout	During rRT	At 36 Gy	98 Gy
9	Right parotid gland	Right parotid gland	rT4bN0	60 months	Brain necrosis	40 months	55 Gy	91 Gy

RTOG; Radiation Therapy Oncology Group, BED_10_; biological equivalent dose.

**Table 4:** Univariable and multivariable analyses of prognostic factors for overall survival and disease-free survival.

Variable	Overall Survival				Disease-Free Survival			
	Univariate		Multivariate		Univariate		Multivariate	
	HR (95% CI)	*p* Value	HR (95% CI)	*p* Value	HR (95% CI)	*p* Value	HR (95% CI)	*p* Value
Age > 52 vs. ≤52	4.55 (2.01–10.32)	**0.01**	5.122 (1.901–13.804)	**0.01**	1.717 (0.846–3.481)	0.13		
Male vs. female	3.32 (1.15–9.57)	**0.02**	3.165 (0.922–10.869)	0.07	2.24 (0.97–5.17)	**0.05**	0.1218 (2.050–0.826)	0.09
PS 0 vs. PS 1–2	2.92 (1.36–6.27)	**0.01**	2.800 (1.402–5.590)	**0.01**	2.46 (1.26–4.81)	**0.01**	2.8 (1.402–5.590)	**0.01**
rT0–2 vs. rT3–4	1.01 (0.49–2.09)	0.98			0.97 (0.50–1.88)	0.93		
rN1–3 vs. 0	1.41 (0.67–2.98)	0.36			1.31 (0.67–2.59)	0.43		
NPC vs. larynx	0.23 (0.07–0.79)	**0.01**	0.286 (0.075–1.086)	0.07	0.42 (0.15–1.20)	0.33		
Oral cavity vs. larynx	1.43 (0.54–3.77)		1.287 (0.481–3.444)	0.62	1.20 (0.47–3.04)			
Salivary gland vs. larynx	0.35 (0.11–1.11)		0.417 (0.118–1.476)	0.18	0.67 (0.25–1.76)			
Skin vs. larynx	0.36 (0.09–1.39)		0.191 (0.041–0.888)	**0.04**	0.72 (0.22–2.36)			
Adjuvant RT vs. adjuvant CRT	0.42 (0.13–1.37)	0.16			0.95 (0.36–2.50)	0.81		
CRT vs. adjuvant CRT	0.44 (0.18–1.11)				0.81 (0.35–1.86)			
Definitive RT vs. adjuvant CRT	1.03 (0.37–2.86)				1.30 (0.47–3.61)			
Others vs. conventional fractionation	1.08 (0.25–4.60)	0.92			1.09 (0.33–3.60)	0.88		
rRT BED_10_ rRT > 60 vs. rRT ≤ 60	0.52 (0.20–1.36)	0.18			0.37 (0.14–0.94)	**0.03**	0.341 (0.126–0.919)	**0.03**
Time since prior RT > 2 years vs. ≤years	0.66 (0.28–1.58)	0.35			0.70 (0.31–1.54)	0.37		
Concurrent chemotherapy yes vs. no	1.05 (0.48–2.30)	0.89			0.88 (0.44–1.76)	0.72		

Significant *p*-values in bold. HR indicates hazard ratio; CI, confidence interval; PS, performance status; rRT, re-irradiation; BED, biological equivalent dose; NPC, nasopharyngeal carcinoma; RT, radiotherapy; CRT concurrent chemoradiation.

## Data Availability

Data is unavailable due to ethical restrictions.
